# Sexual dimorphism and age effects in CKD and type 2 diabetes in the CONFIDENCE trial

**DOI:** 10.1093/ndt/gfag075

**Published:** 2026-03-27

**Authors:** Rajiv Agarwal, Jennifer B Green, Hiddo J L Heerspink, Johannes F E Mann, Janet B McGill, Amy K Mottl, Masaomi Nangaku, Julio Rosenstock, Muthiah Vaduganathan, Charlie Scott, Li Li, Na Li, Carolina Aldworth, Meike Brinker, Peter Rossing

**Affiliations:** Division of Nephrology, Richard L. Roudebush VA Medical Center & Indiana University School of Medicine, Indianapolis, IN, USA; Division of Endocrinology, Department of Medicine and Duke Clinical Research Institute, Duke University School of Medicine, Durham, NC, USA; Department of Clinical Pharmacy and Pharmacology, University Medical Center Groningen, University of Groningen, Groningen, The Netherlands; KfH Kidney Centre, Munich, Germany; Department of Nephrology & Hypertension, Friedrich Alexander University, Erlangen, Germany; Division of Endocrinology, Metabolism & Lipid Research, Washington University in St. Louis, School of Medicine, St. Louis, MO, USA; University of North Carolina Kidney Center, UNC School of Medicine, Chapel Hill, NC, USA; Division of Nephrology and Endocrinology, The University of Tokyo Graduate School of Medicine, Tokyo, Japan; Velocity Clinical Research at Medical City, Dallas, TX, USA; Division of Cardiovascular Medicine, Brigham and Women’s Hospital and Harvard Medical School, Boston, MA, USA; Clinical Statistics and Analytics, Bayer US LLC, Whippany, NJ, USA; Global Medical Evidence, Bayer AG, Berlin, Germany; Clinical Development and Operation, Bayer Healthcare, Beijing, China; US Medical Affairs, Bayer US LLC, Whippany, NJ, USA; Cardiology and Nephrology Clinical Development, Bayer AG, Berlin, Germany; Steno Diabetes Center Copenhagen, Copenhagen, Denmark; Department of Clinical Medicine, University of Copenhagen, Copenhagen, Denmark

**Keywords:** albuminuria, chronic renal insufficiency, diabetes mellitus type 2, empagliflozin, finerenone, mineralocorticoid receptor antagonist, SGLT2 inhibitors, sodium-glucose transporter 2 inhibitor

## Abstract

**Introduction:**

Concurrent initiation of finerenone and empagliflozin can lower albuminuria more than component therapies at Day 180 in participants with chronic kidney disease (CKD) and type 2 diabetes (T2D ). We evaluated baseline clinical characteristics and longitudinal changes in urinary albumin-to-creatinine ratio (UACR) across age and sex cohorts.

**Methods:**

In CONFIDENCE (NCT05254002), participants with T2D, CKD (estimated glomerular filtration rate [eGFR] 30–90 ml/min/1.73 m^2^), and albuminuria (UACR 100 to <5000 mg/g) on stable doses of renin–angiotensin system inhibitors were randomized 1:1:1 to empagliflozin 10 mg/day, finerenone 10 or 20 mg/day, or both. We stratified participants by age quartiles (≤60 years, 61–68 years, 69–74 years , >74 years) and sex. Outcomes included % change in UACR from baseline over time and incidences of key adverse events.

**Results:**

Increasing age was associated with higher baseline prevalence of atherosclerotic cardiovascular disease and lower eGFR. Older age was a significant predictor of UACR reduction, with –10.2% (95% CI: –15.5, –4.6) incremental change per 10-year age increase at Day 180 (*P* for age effect = 0.001). Women exhibited an 18.6% (95% CI: 6.3, 29.2) greater UACR reduction at Day 180 than men (*P* for sex effect = 0.008). These effects were independent of treatment; there was no interaction between age (*P*-interaction = 0.28) or sex (*P*-interaction = 0.46) on combination therapy treatment effect. Safety outcomes were consistent across all demographics.

**Conclusions:**

The UACR-lowering response of finerenone and empagliflozin scaled linearly with age (10% per decade) and sex (∼20% more among women). The benefit of combination therapy was independent of age and sex.

## Graphical Abstract

**Graphical Abstract:**
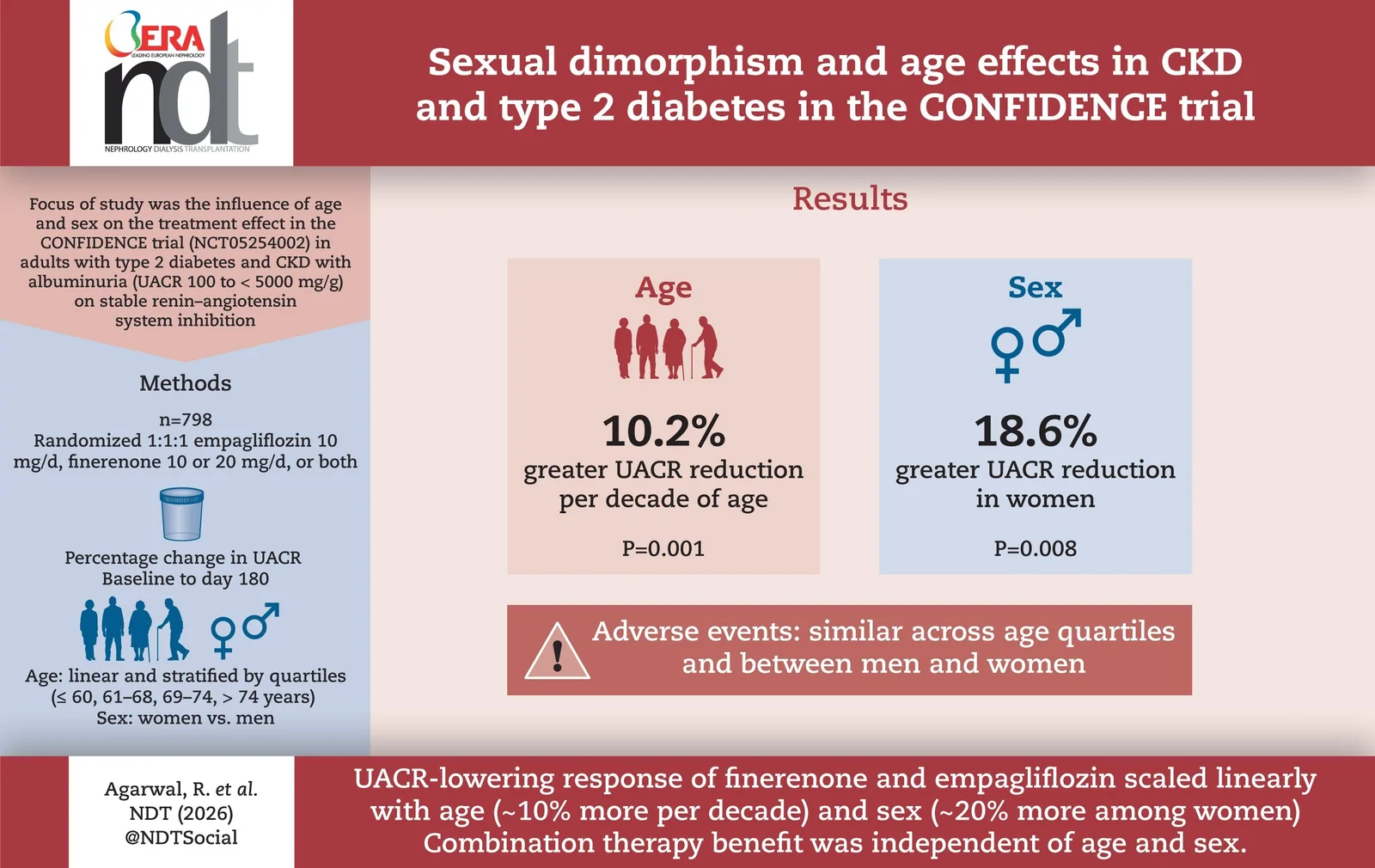

KEY LEARNING POINTS **What was known:**

- Women with type 2 diabetes face a disproportionately greater cardiovascular risk than men, experiencing earlier and more severe deterioration of cardiovascular risk factors. Similarly, increasing age associates with accumulating cardiovascular risk factors and events.
- Simultaneous therapy with finerenone and empagliflozin effectively reduces urinary albumin-to-creatinine ratio (UACR) in patients with chronic kidney disease and type 2 diabetes.
- Despite extensive subgroup analyses in major trials, sex-stratified UACR outcomes remained unreported, leaving it unclear whether women derive similar clinical benefits to men. Rigorous evaluation of continuous longitudinal trajectories of UACR with age remain unknown.

**This study adds:**

- The UACR-lowering response to finerenone, empagliflozin, or their combination scales linearly with advancing age, yielding an incremental 10.2% UACR reduction per 10-year greater age.
- Women exhibited a significantly greater anti-albuminuric response to treatment than men, achieving an 18.6% greater UACR reduction at Day 180.
- These enhanced responses associated with advancing age and female sex were independent of the specific treatment assignment and did not compromise the safety profile.

**Potential impact:**

- The robust efficacy and consistent safety across all age groups emphasize that older individuals with albuminuria should be actively considered for these kidney-protective treatments.
- The superior treatment response in women suggests their greater increase in cardiovascular and kidney risk may result from suboptimal clinical practice and undertreatment, rather than biological nonresponse.
- These findings establish a novel mechanistic framework for personalized kidney care, highlighting the clinical necessity of factoring intersectional biology into therapeutic expectations.

## INTRODUCTION

Diabetes confers a disproportionately greater cardiovascular risk in women than in men. Women with diabetes have approximately two-fold excess coronary heart disease risk compared with men with diabetes, and a three-fold excess fatal coronary heart disease risk compared with women without diabetes [1]. The American Heart Association reports that women with type 2 diabetes experience earlier onset of, and higher mortality from, myocardial infarction than men [1]. Also, a meta-analysis found that the pooled relative risk for cardiovascular disease mortality in individuals with diabetes was 2.42 in women versus 1.86 in men compared with those without diabetes [2,3]. The presence of chronic kidney disease (CKD) in those with type 2 diabetes further magnifies cardiovascular risk [4,5]. However, it is unclear whether women derive similar or greater benefits with treatment than men.

The Health and Retirement Study reported that an earlier onset of type 2 diabetes (i.e. at age 50–59 years) significantly increased the risk of heart disease and stroke compared with no diabetes in the same age group [6]. Notably, the cardiovascular risks associated with diabetes decreased progressively with advancing age at diagnosis, suggesting that younger adults face a more aggressive disease course than older adults [6]. In a meta-analysis of data from more than 80 000 participants aged ≥65 years enrolled in five prospective studies, presence versus absence of type 2 diabetes at baseline was associated with increased risk of cardiovascular events and all-cause mortality [7]. Elsewhere, duration of disease rather than patient age has been linked to microvascular complications of diabetes, such as kidney failure [8].

Current treatments for slowing the progression of CKD and reducing the risk of cardiovascular complications in people with CKD and type 2 diabetes include renin–angiotensin system blockers, sodium–glucose cotransporter 2 inhibitors (SGLT2is), the nonsteroidal mineralocorticoid receptor antagonist finerenone, and glucagon-like peptide-1 receptor agonists [9]. In the CONFIDENCE trial (NCT05254002), simultaneous therapy with finerenone and the SGLT2i empagliflozin reduced urinary albumin-to-creatinine ratio (UACR) at Day 180 more than either treatment alone in participants with CKD and type 2 diabetes [10].

In this prespecified analysis of CONFIDENCE trial data, we investigated whether age and sex influenced treatment response with finerenone, empagliflozin, or their combination in participants with type 2 diabetes and CKD.

## MATERIALS AND METHODS

### Study design and population

CONFIDENCE (NCT05254002) was a phase 2, double-blind, randomized controlled trial conducted across 14 countries [11,12]. The trial adhered to the ethical principles derived from the Declaration of Helsinki and received approval from institutional review boards at all sites. All participants provided written informed consent prior to enrollment. The protocol, design, baseline characteristics, and primary results of this trial have been previously published [10–12].

### Eligibility criteria

The study enrolled participants with type 2 diabetes with glycated hemoglobin <11% and CKD, characterized by an estimated glomerular filtration rate (eGFR) of 30–90 ml/min/1.73 m^2^, albuminuria (defined as UACR between 100 and <5000 mg/g measured via the mean value from three first morning void samples), and receiving a stable, maximally tolerated dose of an angiotensin-converting enzyme inhibitor or angiotensin receptor blocker at baseline.

Patients were excluded if they met any of the following criteria: type 1 diabetes, screening potassium >4.8 mmol/l; symptomatic heart failure with reduced ejection fraction, or a recent (within 90 days) stroke, myocardial infarction, or hospitalization for heart failure; or use of an SGLT2i or potassium-binding agent within 8 weeks of screening.

### Randomized treatment and masking

Participants were randomized in a 1:1:1 ratio to receive empagliflozin 10 mg/day, finerenone 10 or 20 mg/day, or their simultaneous combination. Finerenone was initiated at 20 mg/day in participants with a baseline eGFR ≥60 ml/min/1.73 m^2^ or 10 mg/day for those with an eGFR <60 ml/min/1.73 m^2^. Randomization was stratified by eGFR (<60 vs. ≥60 ml/min/1.73 m^2^) and UACR (≤850 vs. >850 mg/g) at screening. To ensure strict blinding, a robust double-blind design was employed, and group assignments remained concealed from investigators, treating physicians, participants, and outcome assessors throughout the study duration.

### Outcome measures and definitions

The primary efficacy outcome of the trial was the relative change in the log-transformed mean UACR from baseline to Day 180 [12]. This analysis evaluated the effects of age and sex on changes in UACR from baseline for up to 180 days following treatment initiation. To evaluate potential age- and sex-related effects on safety, we report key adverse events, including adverse events of special interest that were prespecified as clinically important by the study investigators and the sponsor: symptomatic hypotension and acute kidney injury.

### Statistical analysis

Analysis of the effect of age and sex on the treatment response with finerenone, empagliflozin, or their combination in CONFIDENCE was prespecified. To estimate determinants of the mean change in UACR from baseline, a linear mixed-effects model was used [13]. Fixed effects included the treatments (finerenone, empagliflozin, or their combination), visit (modelled as a discrete variable), treatment-by-visit interaction, region, stratification factors (i.e. eGFR and UACR), age, and sex (details provided below). Random effects included subject and visits. Maximal likelihood estimation was used to derive the estimated means and standard errors. To facilitate interpretation and data visualization, results are presented as marginal means from the linear mixed-effects models with the covariates modeled at their mean values.

To assess the effect of age and sex on UACR, we added age, sex, age-by-visit, and sex-by-visit interaction to the model. Age was analyzed both as a categorical variable, defined by baseline quartiles (including quartile-by-visit interactions), and as a continuous variable using linear mixed-effects models. The linear mixed-effects models assumed a linear relationship between age and UACR. The age quartile analysis was used as a sensitivity analysis to test the robustness of the linear assumption. Similar models were fitted to evaluate the effects of sex.

To assess effect modification by age and sex, we conducted a sensitivity analysis using a model that included interactions between treatment and these variables.

Although age and sex were prespecified, these analyses are exploratory and the *P* values reported lack formal adjustment for multiplicity of comparisons.

## RESULTS

### Baseline demographics and clinical characteristics

Baseline characteristics were analyzed by age quartiles (Q1 ≤ 60 years, Q2 61–68 years, Q3 69–74 years, Q4 > 74 years; Table 1) and sex (Table 2).

**Table 1:** Baseline characteristics by age quartiles.

	Age quartile					
Clinical characteristic	Q1	Q2	Q3	Q4		All participants
	(≤60 years)	(61–68 years)	(69–74 years)	(>74 years)		
Sample size, *n* (%)	201 (25)	223 (28)	190 (24)	184 (23)		798 (100)
Treatment group, *n* (%)						
Combination	62 (31)	65 (29)	70 (37)	71 (39)		268 (34)
Finerenone	74 (37)	79 (35)	58 (31)	53 (29)		264 (33)
Empagliflozin	65 (32)	79 (35)	62 (33)	60 (33)		266 (33)
Age, years, mean (SD)	52.5 (7.1)	64.8 (2.2)	71.5 (1.7)	78.5 (3.1)		66.5 (10.3)
Male sex, *n* (%)	148 (74)	161 (72)	148 (78)	143 (78)		600 (75)
Geographic region, *n* (%)						
North America	54 (27)	61 (27)	48 (25)	62 (34)		225 (28)
Europe	19 (9)	67 (30)	67 (35)	61 (33)		214 (27)
Asia	128 (64)	95 (43)	75 (39)	61 (33)		359 (45)
Body mass index, kg/m^2^, mean (SD)	29.3 (6.0)	29.7 (6.6)	29.5 (6.0)	28.6 (5.4)		29.3 (6.1)
Serum potassium value, mmol/l, mean (SD)	4.5 (0.4)	4.5 (0.4)	4.5 (0.4)	4.4 (0.4)		4.5 (0.4)
Systolic blood pressure, mmHg, mean (SD)	131.7 (12.5)	135.0 (12.9)	136.3 (13.7)	138.0 (13.6)		135.2 (13.3)
History of atherosclerotic cardiovascular disease, *n* (%)	27 (14)	61 (27)	70 (37)	65 (36)		223 (28)
eGFR values, ml/min/1.73 m^2^, mean (SD)	57 (19)	56 (17)	52 (15)	51 (16)		54 (17)
KDIGO CKD stage, *n* (%)						
G1 or G2	82 (41)	89 (40)	55 (29)	49 (27)		275 (35)
G3A	46 (23)	67 (30)	64 (34)	55 (30)		232 (29)
G3B	67 (34)	62 (28)	60 (32)	71 (39)		260 (33)
G4	5 (2)	5 (2)	9 (5)	8 (4)		27 (3)
UACR, mg/g, median (25th, 75th percentiles)	654 (309, 1353)	613 (292, 1158)	566 (274, 1061)	504 (289, 787)		579 (292, 1096)
Severity of albuminuria, *n* (%)						
<300 mg/g	48 (24)	57 (26)	52 (27)	48 (26)		205 (26)
300 to <1000 mg/g	85 (42)	103 (46)	89 (47)	98 (53)		375 (47)
≥1000 mg/g	68 (34)	63 (28)	49 (26)	38 (21)		218 (27)
HbA1c, %, *n* (%)	7.6 (1.4)	7.3 (1.3)	7.1 (1.1)	7.2 (1.0)		7.3 (1.2)
Concomitant medications, *n* (%)^a^						
ACE inhibitor or ARB	198 (99)	219 (99)	186 (99)	182 (99)		785 (99)
Statins	133 (66)	164 (74)	156 (83)	143 (78)		596 (75)
Diuretics	55 (28)	73 (33)	79 (42)	81 (44)		288 (36)
Insulin	78 (39)	93 (42)	71 (38)	75 (41)		317 (40)
GLP-1 RA	45 (22)	50 (23)	42 (22)	44 (24)		181 (23)

a Patients may have been receiving more than one concomitant medication.

ACE, angiotensin-converting enzyme; ARB, angiotensin receptor blocker; CKD, chronic kidney disease; eGFR, estimated glomerular filtration rate; GLP-1 RA, glucagon-like peptide-1 receptor agonist; HbA1c, glycated hemoglobin; KDIGO, Kidney Disease: Improving Global Outcomes; Q, quartile; SD, standard deviation; UACR, urinary albumin-to-creatinine ratio.

**Table 2:** Baseline characteristics by sex.

Clinical characteristic	Women	Men	All participants
Sample size, *n* (%)	198 (25)	600 (75)	798 (100)
Treatment group, *n* (%)			
Combination	67 (34)	201 (34)	268 (34)
Finerenone	61 (31)	203 (34)	264 (33)
Empagliflozin	70 (35)	196 (33)	266 (33)
Age, years, mean (SD)	65.8 (10.2)	66.7 (10.3)	66.5 (10.3)
Male sex, *n* (%)	0 (0)	600 (100)	600 (75)
Geographic region, *n* (%)			
North America	82 (41)	143 (24)	225 (28)
Europe	42 (21)	172 (29)	214 (27)
Asia	74 (37)	285 (48)	359 (45)
Body mass index, kg/m^2^, mean (SD)	29.8 (6.6)	29.1 (5.9)	29.3 (6.1)
Serum potassium value, mmol/l, mean (SD)	4.5 (0.5)	4.5 (0.4)	4.5 (0.4)
Systolic blood pressure, mmHg, mean (SD)	133.9 (14.0)	135.6 (13.0)	135.2 (13.3)
History of atherosclerotic cardiovascular disease, *n* (%)	45 (23)	178 (30)	223 (28)
eGFR values, ml/min/1.73 m^2^, mean (SD)	54 (18)	54 (17)	54 (17)
KDIGO CKD stage, *n* (%)			
G1 or G2	68 (35)	207 (35)	275 (35)
G3A	61 (31)	171 (29)	232 (29)
G3B	61 (31)	199 (33)	260 (33)
G4	6 (3)	21 (4)	27 (3)
UACR, mg/g, median (25th, 75th percentiles)	548 (270, 986)	592 (298, 1176)	579 (292, 1096)
Severity of albuminuria, *n* (%)			
<300 mg/g	56 (28)	149 (25)	205 (26)
300 to <1000 mg/g	96 (48)	279 (46)	375 (47)
≥1000 mg/g	46 (23)	172 (29)	218 (27)
HbA1c, %, mean (SD)	7.4 (1.3)	7.3 (1.2)	7.3 (1.2)
Concomitant medications, *n* (%)			
ACE inhibitor or ARB	196 (99)	589 (99)	785 (99)
Statins	152 (77)	444 (74)	596 (75)
Diuretics	81 (41)	207 (35)	288 (36)
Insulin	84 (42)	233 (39)	317 (40)
GLP-1 RA	49 (25)	132 (22)	181 (23)

ACE, angiotensin-converting enzyme; ARB, angiotensin receptor blocker; CKD, chronic kidney disease; eGFR, estimated glomerular filtration rate; GLP-1 RA, glucagon-like peptide-1 receptor agonist; HbA1c, glycated hemoglobin; KDIGO, Kidney Disease: Improving Global Outcomes; SD, standard deviation; UACR, urinary albumin-to-creatinine ratio.

Significant geographic and clinical variations were observed across age quartiles (Table 1). The proportion of participants ≤60 years of age was greater among those recruited from Asia (36%) compared with North America (24%) and Europe (9%). Increasing age was generally positively correlated with higher blood pressure, a greater prevalence of atherosclerotic cardiovascular disease, and increased use of statins and diuretics. Also, higher age was associated with lower eGFR.

Men were, on average, 1 year older than women (Table 2). Geographic distribution showed fewer male participants were recruited in the United States and European Union than in Asia. Clinically, men exhibited a higher prevalence of atherosclerotic cardiovascular disease and had higher UACR than women.

### UACR response by age and sex

The longitudinal changes in UACR from baseline were evaluated across the 5th, 25th, 50th, 75th, and 90th percentiles of age. Regardless of treatment assignment, increasing age was associated with a progressively greater reduction in UACR from baseline at all observed time points (*P* = 0.001; Fig. 1). Similar results were obtained when results were analyzed by quartiles instead of age as a continuous variable (*P* = 0.009; Fig. 2).

**Figure 1:**
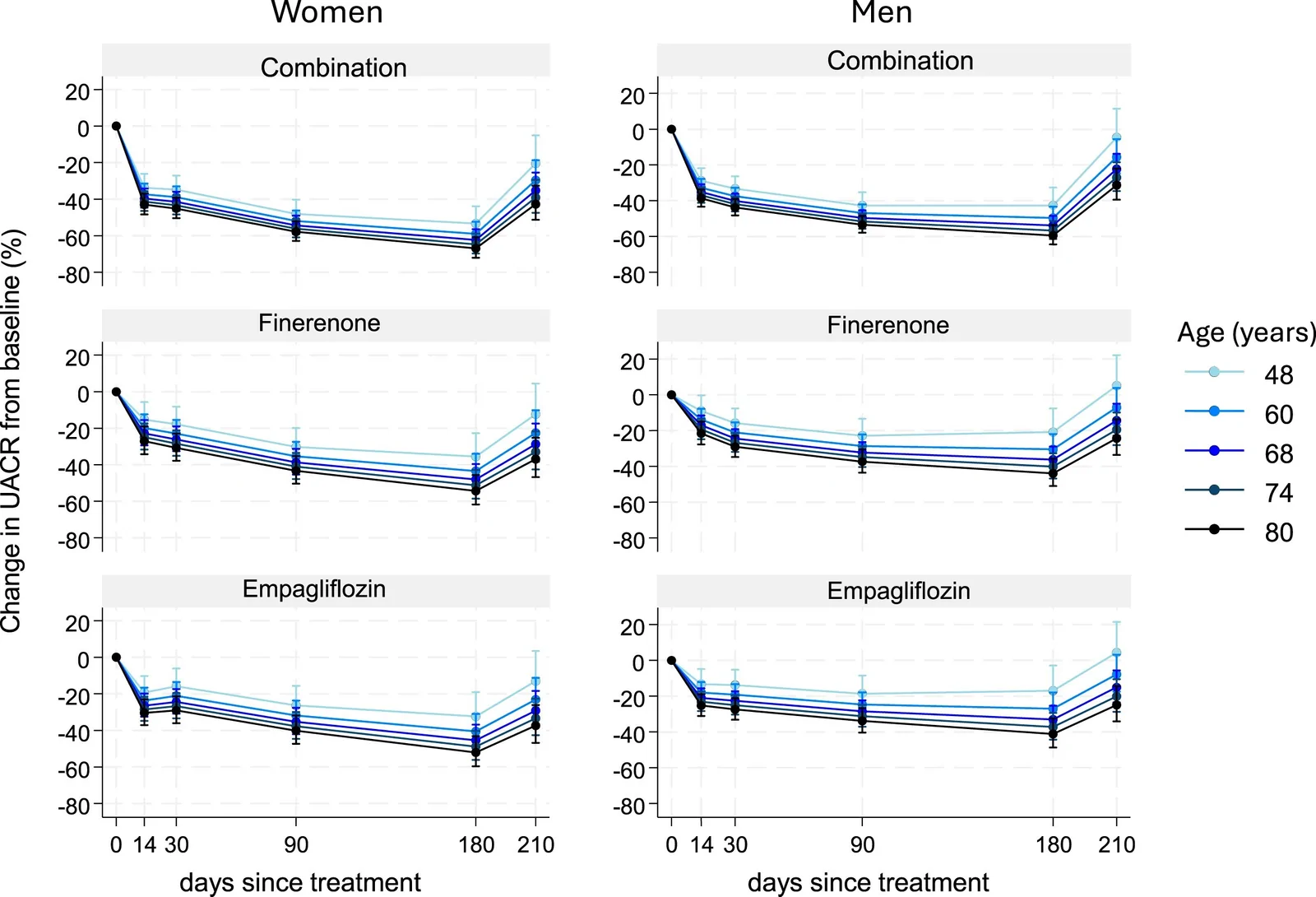
Mean changes in UACR from baseline by treatment group as a function of age and sex. The figure displays the mean changes in UACR at each time point from baseline to Day 210 by treatment. Marginal mean changes were calculated for covariates at ages fixed at 5th, 25th, 50th, 75th, and 95th percentiles (i.e. 48, 60, 68, 74, and 80 years). Error bars represent 95% CI. Treatment was stopped at Day 180; thus, Day 210 represents the UACR 30 days after stopping treatment. CI, confidence interval; UACR, urinary albumin-to-creatinine ratio.

**Figure 2:**
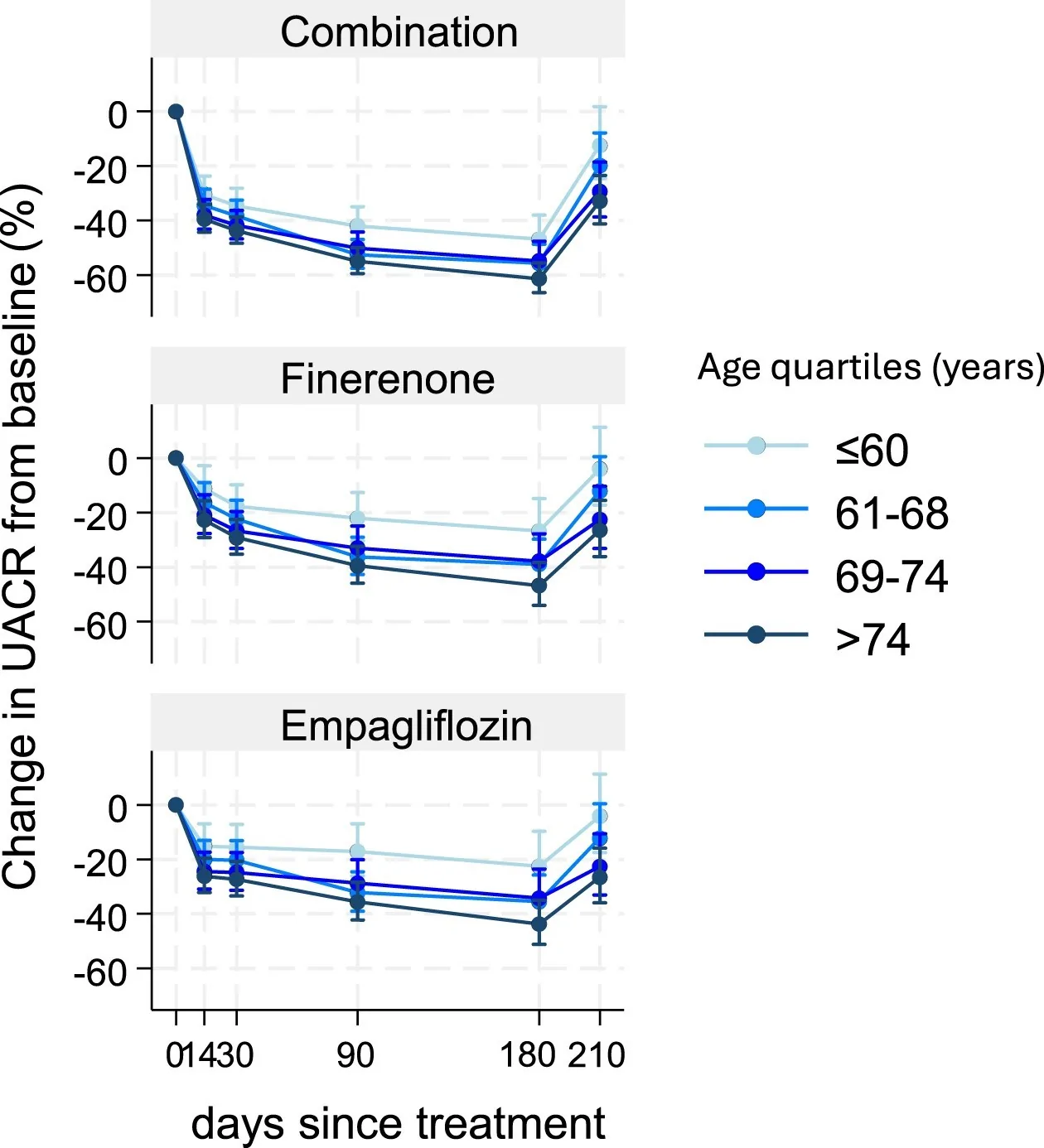
Mean changes in UACR from baseline by treatment group as a function of age quartile. The figure displays the mean changes in UACR at each time point from baseline to Day 210 by treatment. Marginal mean changes were calculated for covariates for each quartile of age. Error bars represent 95% CI. Treatment was stopped at Day 180; thus, Day 210 represents the UACR 30 days after stopping treatment. CI, confidence interval; UACR, urinary albumin-to-creatinine ratio.

Each 10-year increase in age was associated with a ∼5% greater reduction in UACR at Days 14 and 30, and a ∼10% greater reduction at Days 180 and 210 (*P* for age effect = 0.001; Fig. 3). The % change from baseline per 10-year increase in age was –4.5% (95% CI: –7.9, –1.0) on Day 14, –5.2% (95% CI: –8.7, –1.6) on Day 30, –6.3% (95% CI: –10.5, –1.9) on Day 90, –10.2% (95% CI: –15.5, –4.6) on Day 180, and –9.8% (95% CI: –14.9, –4.3) on Day 210. These effects were independent of treatment, with no evidence of interaction between age and the treatment (*P*-interaction = 0.28).

**Figure 3:**
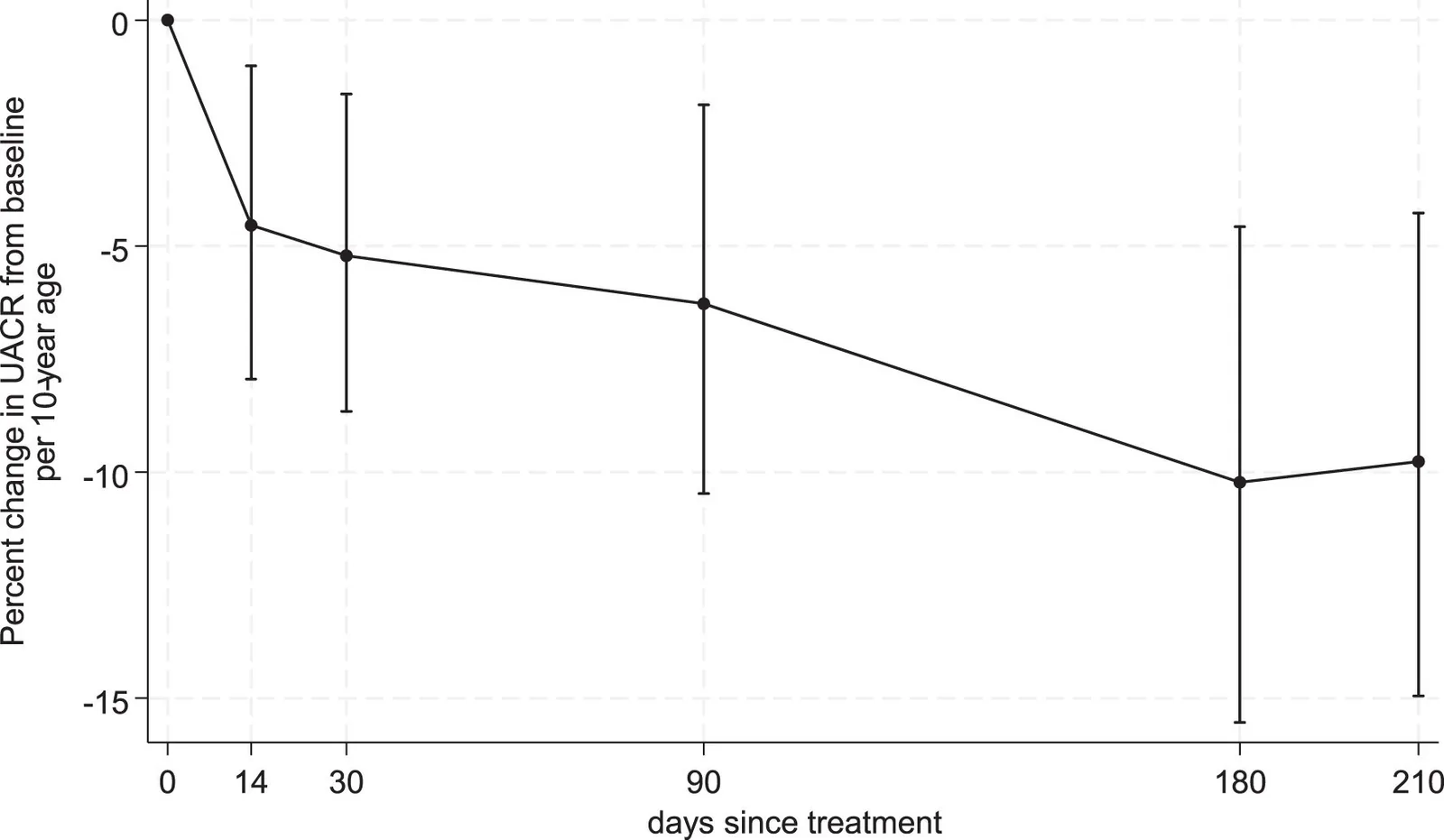
Mean changes in UACR from baseline per 10-year increase in age. The figure displays the mean changes in UACR at each time point from baseline to Day 210 per 10-year increase in age. Marginal mean changes were calculated for covariates at means. Error bars represent 95% CI. Treatment was stopped at Day 180; thus, Day 210 represents the UACR 30 days after stopping treatment. CI, confidence interval; UACR, urinary albumin-to-creatinine ratio.

Treatment response was more pronounced in women than in men. The treatment-effect difference favoring women increased over time. In a fully adjusted model, at Day 180, women demonstrated an 18.6% (95% CI: 6.3, 29.2) greater reduction in UACR compared with men at Day 180 (*P* for sex effect = 0.008) (Table 3). These effects were independent of treatment, with no evidence of interaction between sex and the treatment (*P*-interaction = 0.46).

**Table 3:** Incremental treatment effect in women versus men.

Visit	Change in UACR from baseline (95% CI), %
Day 14	–6.9 (–14.3, 1.1)
Day 30	–2.3 (–10.3, 6.4)
Day 90	–9.5 (–18.6, 0.6)
Day 180	–18.6 (–29.2, –6.3)
Day 210	–16.6 (–27.1, –4.6)

Treatment was stopped at Day 180, thus Day 210 represents the UACR 30 days after stopping treatment.

CI, confidence interval; UACR, urinary albumin-to-creatinine ratio.

### Safety

Safety outcomes were analyzed by age quartiles and sex. No clinically relevant association was observed between age quartiles and the incidence of safety outcomes (Table 4). Also, there was no apparent difference in the safety profile or the frequency of adverse events when comparing men and women (Table 5).

**Table 4:** Safety events by age quartiles.

	Age quartile				
	Q1	Q2	Q3	Q4	All participants
	(≤60 years)	(61–68 years)	(69–74 years)	(>74 years)	
Acute kidney injury, *n*/*N* (%)	0/179 (0.0)	2/212 (0.9)	1/191 (0.5)	1/216 (0.5)	4/798 (0.5)
Combination	0/54 (0.0)	1/60 (1.7)	0/69 (0.0)	1/85 (1.2)	2/268 (0.7)
Finerenone	0/68 (0.0)	1/75 (1.3)	1/61 (1.6)	0/60 (0.0)	2/264 (0.8)
Empagliflozin	0/57 (0.0)	0/77 (0.0)	0/61 (0.0)	0/71 (0.0)	0/266 (0.0)
Genital mycotic infection, *n*/*N* (%)	2/179 (1.1)	4/212 (1.9)	2/191 (1.0)	0/216 (0.0)	8/798 (1.0)
Combination	2/54 (3.7)	1/60 (1.7)	1/69 (1.4)	0/85 (0.0)	4/268 (1.5)
Finerenone	0/68 (0.0)	0/75 (0.0)	0/61 (0.0)	0/60 (0.0)	0/264 (0.0)
Empagliflozin	0/57 (0.0)	3/77 (3.9)	1/61 (1.6)	0/71 (0.0)	4/266 (1.5)
Hyperkalemia, *n*/*N* (%)	24/179 (13.4)	39/212 (18.4)	24/191 (12.6)	26/216 (12.0)	113/798 (14.2)
Combination	8/54 (14.8)	12/60 (20.0)	13/69 (18.8)	7/85 (8.2)	40/268 (14.9)
Finerenone	9/68 (13.2)	19/75 (25.3)	9/61 (14.8)	11/60 (18.3)	48/264 (18.2)
Empagliflozin	7/57 (12.3)	8/77 (10.4)	2/61 (3.3)	8/71 (11.3)	25/266 (9.4)
Moderate hyperkalemia, *n*/*N* (%)	18/179 (10.1)	36/212 (17.0)	22/191 (11.5)	22/216 (10.2)	98/798 (12.3)
Combination	5/54 (9.3)	11/60 (18.3)	12/69 (17.4)	6/85 (7.1)	34/268 (12.7)
Finerenone	8/68 (11.8)	17/75 (22.7)	8/61 (13.1)	10/60 (16.7)	43/264 (16.3)
Empagliflozin	5/57 (8.8)	8/77 (10.4)	2/61 (3.3)	6/71 (8.5)	21/266 (7.9)
Severe hyperkalemia, *n*/*N* (%)	8/179 (4.5)	11/212 (5.2)	6/191 (3.1)	6/216 (2.8)	31/798 (3.9)
Combination	4/54 (7.4)	3/60 (5.0)	3/69 (4.3)	2/85 (2.4)	12/268 (4.5)
Finerenone	2/68 (2.9)	6/75 (8.0)	2/61 (3.3)	2/60 (3.3)	12/264 (4.5)
Empagliflozin	2/57 (3.5)	2/77 (2.6)	1/61 (1.6)	2/71 (2.8)	7/266 (2.6)
Symptomatic hypotension, *n*/*N* (%)	0/179 (0.0)	0/212 (0.0)	3/191 (1.6)	0/216 (0.0)	3/798 (0.4)
Combination	0/54 (0.0)	0/60 (0.0)	3/69 (4.3)	0/85 (0.0)	3/268 (1.1)
Finerenone	0/68 (0.0)	0/75 (0.0)	0/61 (0.0)	0/60 (0.0)	0/264 (0.0)
Empagliflozin	0/57 (0.0)	0/77 (0.0)	0/61 (0.0)	0/71 (0.0)	0/266 (0.0)
Urosepsis, *n*/*N* (%)	1/179 (0.6)	0/212 (0.0)	1/191 (0.5)	0/216 (0.0)	2/798 (0.3)
Combination	1/54 (1.9)	0/60 (0.0)	0/69 (0.0)	0/85 (0.0)	1/268 (0.4)
Finerenone	0/68 (0.0)	0/75 (0.0)	0/61 (0.0)	0/60 (0.0)	0/264 (0.0)
Empagliflozin	0/57 (0.0)	0/77 (0.0)	1/61 (1.6)	0/71 (0.0)	1/266 (0.4)
eGFR drop ≥30% at Day 30, *n*/*N* (%)	4/179 (2.2)	11/212 (5.2)	10/191 (5.2)	5/216 (2.3)	30/798 (3.8)
Combination	3/54 (5.6)	6/60 (10.0)	5/69 (7.2)	3/85 (3.5)	17/268 (6.3)
Finerenone	1/68 (1.5)	3/75 (4.0)	4/61 (6.6)	2/60 (3.3)	10/264 (3.8)
Empagliflozin	0/57 (0.0)	2/77 (2.6)	1/61 (1.6)	0/71 (0.0)	3/266 (1.1)

eGFR, estimated glomerular filtration rate; Q, quartile.

**Table 5:** Safety events by sex.

	Sex		All participants
	Women	Men	
Acute kidney injury, *n*/*N* (%)	3/198 (1.5)	1/600 (0.2)	4/798 (0.5)
Combination	2/67 (3.0)	0/201 (0.0)	2/268 (0.7)
Finerenone	1/61 (1.6)	1/203 (0.5)	2/264 (0.8)
Empagliflozin	0/70 (0.0)	0/196 (0.0)	0/266 (0.0)
Genital mycotic infection, *n*/*N* (%)	4/198 (2.0)	4/600 (0.7)	8/798 (1.0)
Combination	1/67 (1.5)	3/201 (1.5)	4/268 (1.5)
Finerenone	0/61 (0.0)	0/203 (0.0)	0/264 (0.0)
Empagliflozin	3/70 (4.3)	1/196 (0.5)	4/266 (1.5)
Hyperkalemia, *n*/*N* (%)	22/198 (11.1)	91/600 (15.2)	113/798 (14.2)
Combination	5/67 (7.5)	35/201 (17.4)	40/268 (14.9)
Finerenone	9/61 (14.8)	39/203 (19.2)	48/264 (18.2)
Empagliflozin	8/70 (11.4)	17/196 (8.7)	25/266 (9.4)
Moderate hyperkalemia, *n*/*N* (%)	19/198 (9.6)	79/600 (13.2)	98/798 (12.3)
Combination	4/67 (6.0)	30/201 (14.9)	34/268 (12.7)
Finerenone	8/61 (13.1)	35/203 (17.2)	43/264 (16.3)
Empagliflozin	7/70 (10.0)	14/196 (7.1)	21/266 (7.9)
Severe hyperkalemia, *n*/*N* (%)	6/198 (3.0)	25/600 (4.2)	31/798 (3.9)
Combination	2/67 (3.0)	10/201 (5.0)	12/268 (4.5)
Finerenone	2/61 (3.3)	10/203 (4.9)	12/264 (4.5)
Empagliflozin	2/70 (2.9)	5/196 (2.6)	7/266 (2.6)
Symptomatic hypotension, *n*/*N* (%)	1/198 (0.5)	2/600 (0.3)	3/798 (0.4)
Combination	1/67 (1.5)	2/201 (1.0)	3/268 (1.1)
Finerenone	0/61 (0.0)	0/203 (0.0)	0/264 (0.0)
Empagliflozin	0/70 (0.0)	0/196 (0.0)	0/266 (0.0)
Urosepsis, *n*/*N* (%)	2/198 (1.0)	0/600 (0.0)	2/798 (0.3)
Combination	1/67 (1.5)	0/201 (0.0)	1/268 (0.4)
Finerenone	0/61 (0.0)	0/203 (0.0)	0/264 (0.0)
Empagliflozin	1/70 (1.4)	0/196 (0.0)	1/266 (0.4)
eGFR drop ≥30% at Day 30, *n*/*N* (%)	9/198 (4.5)	21/600 (3.5)	30/798 (3.8)
Combination	6/67 (9.0)	11/201 (5.5)	17/268 (6.3)
Finerenone	1/61 (1.6)	9/203 (4.4)	10/264 (3.8)
Empagliflozin	2/70 (2.9)	1/196 (0.5)	3/266 (1.1)

eGFR, estimated glomerular filtration rate.

## DISCUSSION

In this prespecified analysis of the CONFIDENCE trial, we found that advancing age was associated with an increasing prevalence of atherosclerotic cardiovascular disease and lower eGFR at baseline. However, older age was a significant predictor of treatment-associated UACR reduction, with a ∼10% incremental decrease per 10-year increase in age by the end of treatment at Day 180. Women exhibited ∼20% greater UACR reduction than men at the same time point. These changes were independent of treatment (finerenone, empagliflozin, or the combination). Safety outcomes were consistent across all age quartiles and both sexes. Thus, advancing age and female sex were associated with an enhanced UACR-lowering response to finerenone and/or empagliflozin without evidence of a compromised safety profile.

The observation that women demonstrated a significantly more robust response to treatment than men (∼20% greater reduction in UACR at Day 180) represents a key finding for personalized kidney care. The mechanisms underlying sex differences in cardiovascular risk among people with type 2 diabetes are complex. While it is well recognized that sex hormones play important roles, their effects are multifaceted—estrogens are not uniformly cardioprotective, nor are androgens uniformly detrimental, particularly in individuals with diabetes [14]. Exactly how sex dimorphism influenced treatment response, regardless of finerenone, empagliflozin, or their combination, remains unclear.

There are no published post-hoc or subgroup analyses from major SGLT2i trials that specifically report sex-stratified UACR outcomes. Likewise, extensive pooled and meta-analyses that included data from EMPA-REG OUTCOME, CANVAS, DECLARE-TIMI 58, CREDENCE, and DAPA-CKD, as well as other key trials, do not include sex-specific albuminuria data, despite comprehensive subgroup analyses by baseline eGFR and UACR categories [15–17]. Although the treatment effect of finerenone is not modified, a FIDELITY post-hoc analysis demonstrated that women appear to experience a greater anti-albuminuric effect than men, regardless of placebo or finerenone administration [18].

Diabetes disproportionately increases cardiovascular risk in women, effectively neutralizing their biological cardioprotection [1–3]. This disparity stems from a more rapid and severe deterioration of metabolic markers—including BMI, blood pressure, and inflammation—as women transition from healthy glucose levels to diabetes [19,20]. Furthermore, clinical data from the CONFIDENCE trial indicate that while women respond more effectively to albuminuria-lowering treatments than men, they remain victims of undertreatment [1]. This suggests that the poorer outcomes observed in women are likely due to systemic issues like underdiagnosis and suboptimal clinical management rather than a lack of biological response to therapy.

There appears to be a linear relationship between advancing age and the magnitude of UACR reduction. Our results suggest that for every decade of increased age, clinicians can expect an additional 10% reduction in albuminuria at 6 months. Although the treatment effect of finerenone is not modified by age, the aforementioned FIDELITY analysis demonstrated that with increasing age there appeared to be a greater anti-albuminuric effect with finerenone administration [18]. Findings from the Health and Retirement Study data suggest a greater cardiovascular risk when diabetes is diagnosed in people aged in their 50s compared with age-matched controls without diabetes [6]. Utilizing the dual-mechanism approach of combining SGLT2is with a nonsteroidal mineralocorticoid receptor antagonist early in the disease course (specifically, for individuals diagnosed at the relatively young age of 50–59 years) likely offers the greatest “lifetime” benefit in slowing the progression of both CKD and its associated cardiovascular complications. The CONFIDENCE trial shows that the UACR reduction is even greater at an older age. Accordingly, older individuals with albuminuria should be considered for treatment to help prevent both kidney and cardiovascular outcomes in this population.

The consistent safety profile across all age quartiles is reassuring. In geriatric medicine, a major concern is the “prescribing paradox”—where those who could benefit most (i.e. older people with multiple comorbidities) are more likely to experience prescribing omissions, potentially related to concerns about polypharmacy and drug–drug interactions [21,22]. These results suggest that the treatment remains safe even as eGFR declines and age-related comorbidities increase.

While observed correlations between sex, age, and clinical outcomes should not be interpreted as inherently causal, these findings warrant further mechanistic investigation. Specifically, future research should evaluate whether the heightened therapeutic response in women is driven by sex-specific variations in tubuloglomerular feedback sensitivity during SGLT2 inhibition or differential mineralocorticoid receptor activation within podocytes and tubular cells. Additionally, the roles of estrogen receptor-mediated modulation of albumin receptor trafficking and hormone-independent effects of sex-chromosome complements on transporter regulation remain critical areas for exploration [23,24].

There are some limitations of the current study. For example, only 25% of the study population were women and follow-up was short. Also, variation in UACR is substantial and the follow-up period on treatment was limited to 180 days. Finally, the *P* values were not adjusted for multiplicity. Nonetheless, these analyses underscore a critical gap; major SGLT2i trials have not reported sex-stratified UACR outcomes despite comprehensive subgroup analyses by other variables. To our knowledge, these data provide the first evidence identifying clinically meaningful sex differences in treatment response.

In conclusion, the 20% greater UACR reduction in women and the additional 10% reduction per decade of age, seen consistently with finerenone, empagliflozin, and their combination therapy, suggest fundamental biological differences in kidney response to injury and treatment rather than drug-specific effects. The relative efficacy and safety across all age and sex cohorts suggests that these therapies are useful across a broad age range and in both sexes to enhance kidney and cardiovascular protection.

## Data Availability

Availability of the data underlying this publication will be determined according to Bayer’s commitment to the EFPIA/PhRMA “Principles for responsible clinical trial data sharing.” This pertains to scope, timepoint, and process of data access. As such, Bayer commits to sharing upon request from qualified scientific and medical researchers, patient-level clinical trial data, study-level clinical trial data, and protocols from clinical trials in patients for medicines and indications approved in the United States (US) and European Union (EU) as necessary for conducting legitimate research. This applies to data on new medicines and indications that have been approved by the EU and US regulatory agencies on or after 1 January 2014. Interested researchers can use www.vivli.org to request access to anonymized patient-level data and supporting documents from clinical studies to conduct further research that can help advance medical science or improve patient care. Information on the Bayer criteria for listing studies and other relevant information is provided in the member section of the portal. Data access will be granted to anonymized patient-level data, protocols and clinical study reports after approval by an independent scientific review panel. Bayer is not involved in the decisions made by the independent review panel. Bayer will take all necessary measures to ensure that patient privacy is safeguarded.
